# COVID-19 Pandemic: Huge Stress Test for Health System Could Be a Great Opportunity to Update the Workflow in a Modern Surgical Pathology

**DOI:** 10.3390/cancers13133283

**Published:** 2021-06-30

**Authors:** Antonino Belfiore, Giovanni Centonze, Patrick Maisonneuve, Carla Riva, Daniele Morelli, Alessandro Mangogna, Giovanna Sabella, Giancarlo Pruneri, Massimo Milione

**Affiliations:** 1 Second Pathology Unit, Department of Pathology and Laboratory Medicine, Fondazione IRCCS Istituto Nazionale dei Tumori Milano, 20133 Milan, Italy; antonino.belfiore@istitutotumori.mi.it; 2 First Pathology Unit, Department of Pathology and Laboratory Medicine, Fondazione IRCCS Istituto Nazionale dei Tumori Milano, 20133 Milan, Italy; giovanni.centonze@istitutotumori.mi.it (G.C.); carla.riva@istitutotumori.mi.it (C.R.); giovanna.sabella@istitutotumori.mi.it (G.S.); 3 Division of Epidemiology and Biostatistics, European Institute of Oncology IRCCS, IEO, 20141 Milan, Italy; patrick.maisonneuve@ieo.it; 4 Unit of Laboratory Medicine, Department of Pathology and Laboratory Medicine, Fondazione IRCCS Istituto Nazionale dei Tumori Milano, 20133 Milan, Italy; daniele.morelli@istitutotumori.mi.it; 5 Institute for Maternal and Child Health, IRCCS Burlo Garofalo, 34137 Trieste, Italy; alessandro.mangogna@burlo.trieste.it

**Keywords:** SARS-CoV-2, preventive measures, cancer screening, health management

## Abstract

**Simple Summary:**

The COVID-19 pandemic has hit Northern Italy’s regions hard in terms of deaths since February 2020. Containment measures have been applied to avoid contagion and reduce the patient infection rate. In this manuscript, we report the experience of the Pathology Department of the Fondazione IRCCS Istituto Nazionale Tumori in Milan, during the period of the first lockdown that occurred in Lombardy from March to May 2020, focusing on the variation in terms of exams between the pre-COVID-19 and COVID-19 periods and describing the measures applied to guarantee the safeguarding of workers. Moreover, we calculated if changes introduced within the workflow affected the average diagnosis time using Turn-Around-Time (TAT) metrics released by the Lombardy Region. We showed a sharp slowdown in exams during the first wave of COVID-19 and that the measures applied for the safeguarding of the personnel turned out to be feasible and did not affect the overall performance of the Pathology Department.

**Abstract:**

Background: On December 2019, an outbreak of atypical pneumonia, known as COVID-19, was identified in Wuhan, China. This disease, characterized by the rapid human-to-human transmission of the severe acute respiratory syndrome coronavirus 2 (SARS-CoV-2), has spread rapidly in more than 200 countries. Northern Italy’s regions have been hit hard in terms of deaths. Here, we report the experience of the Pathology Department of the Fondazione IRCCS Istituto Nazionale Tumori (INT) in Milan, the first Italian public cancer center, in the period of the lockdown that took place in Lombardy from March to May 2020. Method: The variation in terms of exams was calculated in two different timeframes: December 2019–February 2020 (pre-COVID-19) and March–May 2020 (COVID-19). During these periods, Turn-Around-Time (TAT) metrics released by the Lombardy Region were calculated to assess if changes applied to guarantee the safeguarding of workers affected the average diagnosis time. Results: In the COVID-19 period, there was a decrease for all the performed exams. The most considerable decrease was observed for PAP tests (−81.6%), followed by biopsies (−48.8%), second opinions (−41.7%), and surgical (−31.5%), molecular (−29.4%) and cytological (−18.1%) tests. Measures applied within the Pathology Department, such as digital pathology, remote working, rotations and changes in operating procedures, improved the diagnostic performance as required by the guidelines of the Lombardy Region in terms of TAT. At the same time, the measures applied for the safeguarding of the personnel turned out to be feasible and did not affect the overall performance of the Pathology Department. Conclusions: The sharp slowdown in cancer screening during the first wave of COVID-19 could seriously endanger cancer prevention in the near future.

## 1. Introduction

With over 165 million cases reported and 2 million new cases per day [1], the severe acute respiratory syndrome coronavirus 2 (SARS-CoV-2) pandemic [2,3] has hit Italy particularly hard since March 2020 [4], yielding more than 125,000 deaths in three separate waves, the third of which is currently raging. Since the beginning of the SARS-CoV-2 outbreak, intensive care hospitalization in Lombardy has been very high, urging a profound reorganization of healthcare facilities through the creation of specific COVID-19 hospitals able to guarantee patient isolation and intensive care support, as well as the centralization of treatment for other life-threatening diseases such as cancer in COVID-19-low specialized hubs. In this scenario, Fondazione IRCCS Istituto Nazionale Tumori (INT) in Milan, an Institute of Scientific Hospitalization and Care (IRCCS), was addressed as a hub hospital by the Lombardy Region. INT is the first Italian public cancer center and is one of the main national and international referral centers for cancer treatment. It has 482 beds and is located in a metropolitan area that includes 133 different municipalities and serves more than 3 million people. Considering all these features, INT is in a privileged position to conduct patient care, and preclinical and translational research in oncology. The Department of Pathology has 82 members and is provided with fully equipped labs for routine histopathological assessment and molecular testing. The approach to cancer diagnosis has recently been revolutionized by the introduction of the mutational paradigm, which leverages the comprehensive molecular characterization of tumors for personalized care. Accordingly, we recently applied an innovative organizational model to the INT Department of Pathology, by creating two independent and highly integrated units of surgical and molecular pathology. This structure allowed us to efficiently diagnose and comprehensively profile the case-mix of roughly 50,000 exams per year, either internal or referred by community hospitals, which traditionally represent the core business of our Institution. In this manuscript, we describe the changes in the number of exams and in the workflow of the department units after the first peak of the COVID-19 epidemic in Lombardy, also focusing on the management of biological tissues and procedures to preserve the health of workers.

## 2. Materials and Methods

### 2.1. Study Design

The main objective of this study was to quantify the negative impact of the first SARS-CoV-2 outbreak on cancer patients of our Institution by focusing on the number of exams performed by the INT Department of Pathology. For this reason, we evaluated the number of exams in two different timeframes, i.e., December 2019–February 2020 (pre-COVID-19 period) and March–May 2020 (COVID-19 period). The exams were grouped into different classes—“surgical”, “biopsy”, “second opinion”, “molecular”, “cytology” and “PAP test”—and differentiated by the origins of the exams, namely, (i) day hospital, (ii) inpatients and (iii) outpatients.

During the lockdown period, several to-hospital and within-hospital filters were applied to protect our Institution, as described by Valenza et al. [5]. Accordingly, procedures concerning fresh material handling (fresh sample reduction and processing) and the access of external personnel were modified in both the units of the Department of Pathology, in order to preserve the health of workers [6]. As a secondary aim, we measured the performance of the Pathology Department in diagnosis production, taking advantage of the Turn-Around-Time (TAT) metrics released by the Lombardy Region for the pathology laboratories accredited with the national health system: this procedure establishes that at least 90% of the diagnoses must be carried out within a timeframe pre-specified for each category of exams. To this end, the internal Pathox workflow management (Tesi Elettronica e Sistemi Informativi, Milan, Italy) software was retrospectively interrogated for TAT by comparing both the pre-COVID-19 period and COVID-19 period. Finally, the changes in the operating procedures, applied for the safeguarding of workers, were described as a model for the other Pathology Laboratories.

### 2.2. Operating Procedures and Reorganization of the Working Environment

The main modifications to the operating procedures and working environment are listed below. Table 1 summarizes the changes to each procedure with respect to the pre-COVID situation.

(a) Gross examination

All the samples were already treated in a safe environment using personal protective equipment. Before the SARS-CoV-2 outbreak, sampling procedures were performed on fresh surgery tissues. The first measure adopted was to submit all the specimens to a 24–72 h fixation with 10% buffered formalin. All the fresh surgical materials referred to our department were properly handled by a pathologist, wearing a FFP2 safety mask and single-use sterile gown under a chemical fume cabinet, to improve formalin penetration, and then fixed for 24 h before routine sampling. All the intraoperative examinations were performed wearing FFP2 safe masks and single-use sterile gowns. The cryostat was decontaminated after every procedure using a 95% alcoholic solution.

(b) Cytology

All the instruments dedicated to cytology were secured under a class I biosafety cabinet. Fresh samples, such as bronchoalveolar washes, urine and expectorates were processed by operators wearing FFP2 safe masks and disposable gowns. Fluids (expectorates, Bronchoalveolar lavages and bronchial aspirates) posing a risk of aerosol generation were processed under a laminar flow hood using additional disposables such as FFP2/FFP3 masks. Steps outside the flow hood were always carried out wearing an FFP2/FFP3 mask, glasses or visors, using sealed tubes. As during the pre-COVID-19 period, alcoholic or different fixatives were avoided in order to preserve the integrity of samples and prevent coarctation or flocculation.

(c) Flow cytometry

Sample processing and immunofluorescence labeling were carried out under a laminar flow hood using individual disposables. For the steps performed outside the hood, such as centrifugation, tubes were hermetically sealed. All the instruments were properly sanitized (pipettes and hoods with bleach and common areas with hydroalcoholic solution).

(d) Cytogenetics

Cell cultures from peripheral blood and bone marrow, cell cultures for the analysis of B lymphocyte aspirates, and chromosomal preparations from blood and/or bone marrow aspirates were handled under a sterile laminar flow hood. The operators always wore double gloves, protective glasses or visors and appropriate clothing with additional disposable gowns. Surfaces and centrifuges were sanitized using hydroalcoholic solution.

(e) DNA extraction

DNA extraction from blood samples was always performed using an automated Maxwell extractor (Promega, Milan, Italy). Cartridges were loaded under a laminar flow hood with closed glass. According to the regulations published by the Center for Disease Control and Prevention CDC [7], the operators wore individual protection disposables (surgical masks, a double pair of gloves, protective glasses or visors and a gown). The instruments were sanitized according to the operating instructions. The Maxwell extractor was sanitized internally with UV rays.

(f) Digital Pathology

Slides were digitalized for diagnostic use. Images were acquired on an Aperio ScanscopeXT^®^ (Leica Biosystems Aperio, Wetzlar, Germany) at 40× and 400× magnifications and loaded on a Network Attached Storage (NAS) server in order for them to be accessible and viewable by remotely working pathologists. Software for digital slide viewing and analysis (ImageScope, Aperio, Leica Biosystem) was installed on the pathologist’s personal computer, as well as them being provided with access to the institutional virtual private network (VPN) to work remotely.

### 2.3. Safety of Working Environment

In order to prevent the possible transmission of COVID-19 within the Laboratory Department, several security measures were undertaken. On 8 March 2020, a presidential decree (Law no. 34/2020) established that up to 50% of the staff personnel could be placed in remote working. The decree was then implemented during the second peak of SARS-CoV-2 infections in October 2020, in order to further balance the need to face the pandemic and the continuity of services through flexibility in working hours. We prioritized remote working for the personnel who were actually able to work from home. In particular, pathologists who shared offices with other colleagues were equipped with software for digital slide viewing and VPN access. Accordingly, biologists were also connected to the VPN in order to enable them to use the lab software remotely. These procedures allowed obtaining a 50% reduction in onsite pathologists and biologists, respectively. By contrast, only 15% of the laboratory technician staff were able to carry out remote working, due to the need to manually perform working procedures on site. Rotations were established to guarantee a safe alternation between onsite and remote working. The access of personnel external to the pathology department was restricted to operators carrying samples from surgical rooms and the day hospital. All the offices of the department were already equipped with sensors unlocked through electronic badges, a measure further helping to reduce contacts among inter- and intra-department operators. The reception of patients for pathology report collection or consultation sample delivery was restricted to offices equipped by glass separators, and the opening time was reduced.

### 2.4. Statistical Methods

All the statistical analyses were performed using the R environment for statistical computing and graphics (R Foundation, Vienna, Austria, Version 3.6.2). Comparisons of exams among groups were performed by Fisher’s exact test or the Chi square test for categorical variables. All the tests were two-sided, and *p*-values < 0.05 were considered statistically significant.

## 3. Results

### 3.1. Exam Volume

**Figure 1:**
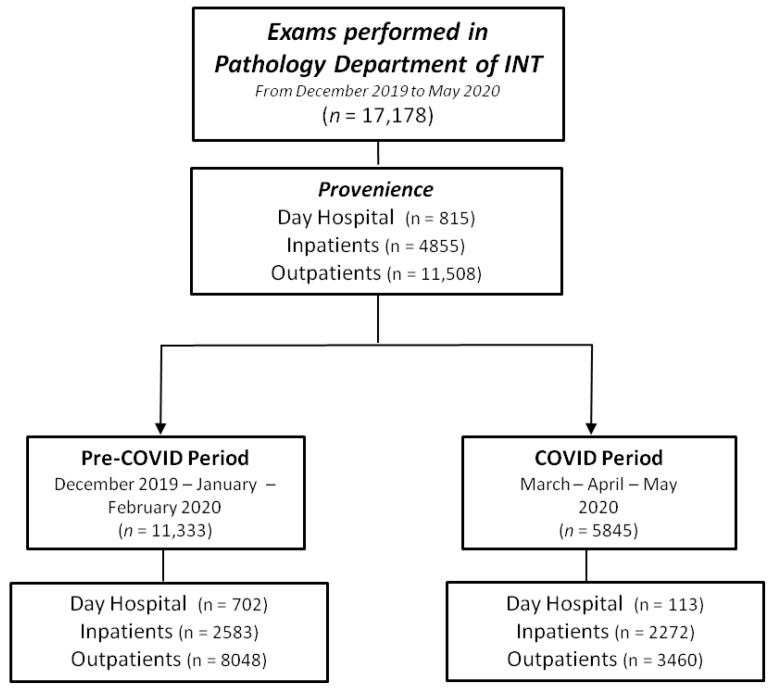
Flowchart of exams performed in pathology department. Abbreviations: INT, Fondazione IRCCS Istituto Nazionale Tumori of Milan (INT).

**Figure 2:**
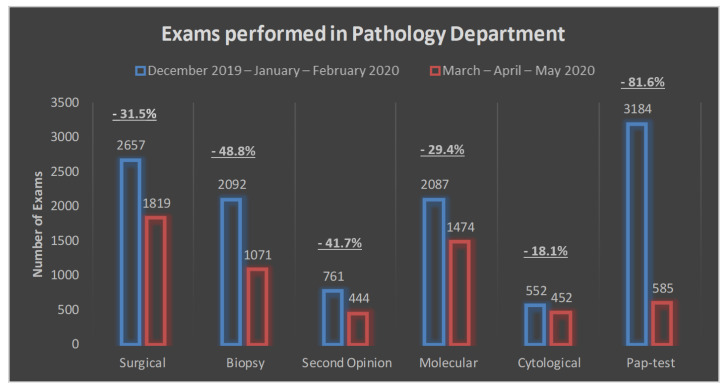
Number of exams performed between the pre-COVID-19 and the COVID-19 period.

The number and distribution of the exams carried out in the department in the pre-COVID-19 and COVID-19 periods are outlined in Figure 1 and Figure 2 and Table 2. Overall, we performed 17,178 exams, distributed as follows: 4476 (26.1%) surgical, 3163 (18.4%) biopsies, 1205 (7.0%) second opinions, 3561 (20.7%) molecular tests, 1004 (5.8%) cytological tests and 3769 (21.9%) PAP tests (Figure 1). Upon the COVID-19 outbreak, there was a decrease for all the performed exams (Figure 2 and Table 2). The most important decrease was observed for PAP tests (−2599 exams, corresponding to a reduction of 81.6%), followed by biopsies (−1021 exams or −48.8%), surgical exams (−838 or −31.5%), molecular tests (−613 or −29.4%), second opinion exams (−317 or −41.7%) and cytological exams (−100 or −18.1%) (Figure 2, Table 2).

### 3.2. Exam Source

During the COVID-19 period, the distribution of the exam sources was modified. Overall, the most important reduction was observed for outpatient exams (−1989 or −40.9%), followed by day hospital exams (−589 or −83.9%) and inpatient exams (−311 or −12.0%). Specifically, the relative frequency of surgical samples and biopsies from the day hospital dropped (19.2% vs. 3.5% and 6.8% vs. 1.7% in the pre-COVID-19 and COVID-19 periods, respectively). Cytological exams also decreased, though to a lesser extent (4.7% vs. 3.5%), while molecular analyses and second opinions were unaltered (1.0% vs. 1.1% and 0.3% vs. 0%, respectively). A similar trend was observed for the outpatients: the surgical (35.1% vs. 32.3%), cytological (46.6% vs. 35.9%), molecular (73.0% vs. 68.9%) and second opinion (61.7% vs. 55.4%) workloads decreased during the COVID-19 period, while biopsies were unchanged (80.8% vs. 80.4%). Finally, we registered an increase in relative frequency in all the exams within the inpatient subgroup during the COVID-19 period.

### 3.3. Turn-Around-Time

The pre-specified TAT was matched for all the test typologies, in both the pre-COVID-19 and COVID-19 quarters under evaluation (Table 3). In particular, a significant increase in TAT exams was observed for surgical (98.2% vs. 95.9%, *p* < 0.0001) and molecular analyses (95.8% vs. 91.8%, *p* < 0.0001) and second opinions (93.5% vs. 85.4%, *p* < 0.0001) in the COVID-19 period compared to the pre-COVID-19 period. On the other hand, no significant TAT changes were observed for biopsies (95.9% vs. 96.2%, *p* = 0.69), cytological exams (97.6% vs. 98.0%, *p* = 0.66) or pap-test exams (100% vs. 100%, *p* = 1.00).

## 4. Discussion

The COVID-19 pandemic keeps challenging health systems worldwide, with repeated infection peaks urging deeply modifying approaches and procedures for safeguarding patients and personnel. During the first lockdown in Lombardy, lasting from March to May 2020, several structures were converted into COVID-19-hospitals, and cancer patients were centralized to a few hubs, including INT Milan. Cancer screening programs including gynecology and mammographic exams were suspended, first and follow-up visits were often postponed, and surgery was delayed except for cases defined as urgent according to specific guidelines released by the regional department of health. In this scenario, cancer centers had the twofold goal of maintaining their proficiency within a COVID-19-low environment through careful screening for COVID-19 infections and new procedures for patient triage as well as internal reorganization [5].

Here, we describe the impact of COVID-19-related procedures (the formalin fixation of fresh tissues, remote working with digital pathology and remote reporting tools) applied upon the first peak of the SARS-CoV-2 outbreak in Lombardy on the TAT of the exams carried out at the units of surgical and molecular pathology in INT Milan. The TAT measures the proficiency of a pathology lab in providing timely diagnoses, a prerequisite for properly applying personalized treatments, especially in cancer patients. We found that the TAT of all the diagnostic procedures evaluated during the COVID-19 period was always >90%; specifically, 93.5–100% of the exams were completed within the prespecified timeframe. This result in spite of a 50% reduction in onsite personnel (mainly pathologists and biologists) and the implementation of time-consuming procedures including 24–72 h formalin fixation for all the samples admitted to the department likely stems from a generalized decrease in the overall workload due to lockdown restrictions. Actually, the volume of histological, cytological and molecular procedures showed a 14.1–36% reduction during the COVID-19 period. The reduction was particularly evident for day-hospital histological and cytological exams, while samples from inpatients subjected to surgery slightly increased, a trend attributable to social confinement with the centralization of cancer patients from general hospitals. PAP tests showed the greatest decrease (−81.6%), further confirming that cancer screening had an abrupt slowdown during the first wave of COVID-19, a trend endangering cancer prevention in the near future. These data are in keeping with a recent report of London J.W. et al. [8], who compared January–April 2019 and 2020 patient data from a network of 20 US institutions accounting for more than 28 million patients, showing a substantial decrease in breast cancer (−89.2%) and colorectal cancer (−84.5%) screenings. Along this line, Maringe et al. [9] predicted a substantial increase in the number of avoidable breast, colorectal, lung and esophageal cancer deaths in England as a result of diagnostic delays due to pandemic lockdown measures in the UK.

We used digital pathology to conjugate personnel safeguarding with diagnostic performance: among the procedural changes made upon the COVID-19 crisis, telemedicine is likely to be maintained and further implemented in the future. Digital pathology allows effectively making diagnoses remotely based on cytological, histological, immunohistochemical and in situ hybridization slides. When properly integrated with the institutional software and the electronical clinical record, this model permits handling all the diagnostic steps following macroscopic examination, eventually enhancing the flexibility of the personnel work schedule.

## 5. Conclusions

Collectively, our data provide evidence that implementing measures for the safeguarding of the personnel is feasible and does not affect the overall performance of the pathology laboratory. Although the model was implemented upon the first lockdown, when a significant reduction in the overall workload was observed, its efficacy was maintained in the second wave of the infection, is likely to be maintained beyond, and may be useful for other pathology laboratories included in a hub and spoke model. Finally, confirming the principle that every crisis represents an opportunity for progress, our data demonstrate that digital pathology should be implemented for a new and more efficient diagnostic approach.

## Figures and Tables

**Table 1:** Summary of the pre- and post-COVID-19 operating procedures modified within the Department of Pathology.

Area Involved	Pre-COVID-19 Period	COVID-19 Period	Maintenance Post COVID-19 Period
Department access	INT personnel admitted	Authorized operators only	Yes
Personnel	100% onsite	50% clinicians onsite	To be considered
		50% biologists onsite	
		85% technicians onsite	
Gross Reduction Lab	Standard PPE for operators	Advanced PPE for operators	To be considered
	Fresh sampling	24–72 h fixation before sampling	No
	Daily intra-operative instrument decontamination	Decontamination after every intra-operative exam	Yes
Cytology Lab	Standard PPE for operators	Advanced PPE for operators	To be considered
	Instruments on bench	Instruments under class I biocabinet	Yes
Flow Cytometry Lab	Standard PPE for operators	Advanced PPE for operators	To be considered
	Daily instrument decontamination	Instrument decontamination between samples	Yes
Cytogenetics Lab	Standard PPE for operators	Advanced PPE for operators	To be considered
	Sample processed under laminal hood	Sample processed under laminal hood	Yes
Molecular Lab	Standard PPE for operators	Advanced PPE for operators	To be considered
	Automatization of nucleic acid extraction	Automatization of nucleic acid extraction under a laminar hood	To be considered
Digital Pathology	For research use only	For diagnostic use	To be considered

**Table 2:** Exams performed in pre-COVID-19 and in COVID-19 quarters.

Exams	Pre-COVID-19 Period (Dec 2019–Feb 2020) *N* (%)	COVID-19 Period (Mar–Apr 2020) *N* (%)	Absolute Change (Relative Change)
**All exams**	11,333 (100)	5845 (100)	−5488 (−48.4%)
**All except pap test**	8149 (71.9)	5260 (90.0)	−2889 (−35.5%)
Inpatients	2583 (31.7)	2272 (43.2)	−311 (−12.0%)
Day hospital	702 (8.6)	113 (2.1)	−589 (−83.9%)
Outpatients	4864 (59.7)	2875 (54.7)	−1989 (−40.9%)
**Surgical**	2657 (23.4)	1819 (31.1)	−838 (−31.5%)
Inpatients	1214 (45.7)	1169 (64.3)	−45 (−3.7%)
Day hospital	511 (19.2)	63 (3.5)	−448 (−88.7%)
Outpatients	932 (35.1)	587 (32.3)	−345 (−63.0%)
**Biopsy**	2092 (18.5)	1071 (18.3)	−1021 (−48.8%)
Inpatients	268 (12.8)	188 (17.6)	−80 (−29.9%)
Day hospital	143 (6.8)	18 (1.7)	−125 (−87.4%)
Outpatients	1681 (80.4)	865 (80.8)	−816 (−51.5%)
**Second opinion**	761 (6.7)	444 (7.6)	−317 (−41.7%)
Inpatients	289 (38.0)	198 (44.6)	−91 (−31.5%)
Day hospital	2 (0.3)	0 (0.0)	−2 (−100%)
Outpatients	470 (61.7)	246 (55.4)	−224 (−47.7%)
**Molecular**	2087 (18.4)	1474 (25.2)	−613 (−29.4%)
Inpatients	543 (26.0)	443 (30.0)	−100 (−18.4%)
Day hospital	20 (1.0)	16 (1.1)	−4 (−20.0%)
Outpatients	1524 (73.0)	1015 (68.9)	−509 (−33.4%)
**Cytological**	552 (4.9)	452 (7.7)	−100 (−18.1%)
Inpatients	269 (48.7)	274 (60.6)	+5 (+1.9%)
Day hospital	26 (4.7)	16 (3.5)	−10 (−38.5%)
Outpatients	257 (46.6)	162 (35.9)	−95 (−37.0%)
Pap test	3184 (28.1)	585 (10.1)	−2599 (−81.6%)

**Table 3:** Turn Around Time of exams performed in Pathology Department.

Exams	Pre-COVID-19 Period (Dec 2019–Feb 2020) *N* (%) in Time	COVID-19 Period (Mar–Apr 2020) *N* (%) in Time	*p*-Value *
Surgical	2549 (95.9)	1786 (98.2)	<0.0001
Biopsies	2013 (96.2)	1027 (95.9)	0.69
Second Opinion	650 (85.4)	415 (93.5)	<0.0001
Molecular	1916 (91.8)	1412 (95.8)	<0.0001
Cytological	541 (98.0)	441 (97.6)	0.66
PAP Test	3184 (100)	585 (100)	1.00

* *p*-Value based on the Fisher exact test; TAT imposed by the INT for 20 working days; Abbreviations: TAT—Turn Around Time.

## Data Availability

The data presented in this study are available on request from the corresponding author.
